# Nontruncating *FRMD7* Variants Are More Penetrant than Truncating Variants in Heterozygous Female Carriers of Infantile Nystagmus

**DOI:** 10.1016/j.xops.2026.101363

**Published:** 2026-08-14

**Authors:** Callum Hunt, Lilian Zevlaris, Benjamin Wong, Gail D.E. Maconachie, Zhanhan Tu, Omar A. Mahroo, Mariya Moosajee, Michel Michaelides, Andrew R. Webster, Jinu Han, Pradeep Vasudevan, Irene Gottlob, Yolanda Markaki, Mervyn G. Thomas

**Affiliations:** 1 The University of Leicester Ulverscroft Eye Unit, School of Psychology and Vision Sciences, University of Leicester, Leicester, United Kingdom; 2 Division of Ophthalmology and Orthoptics, The University of Sheffield, Sheffield, United Kingdom; 3 Moorfields Eye Hospital NHS Foundation Trust, London, United Kingdom; 4 UCL Institute of Ophthalmology, University College London, London, United Kingdom; 5 Department of Ophthalmology, Yonsei University College of Medicine, Seoul, South Korea; 6 Department of Clinical Genetics, University Hospitals of Leicester NHS Trust, Leicester, United Kingdom; 7 Department of Neurology, Cooper University Health Care, Camden, New Jersey; 8 Department of Molecular and Cell Biology, Institute for Structural and Chemical Biology, University of Leicester, Leicester, United Kingdom; 9 Department of Ophthalmology, University Hospitals of Leicester NHS Trust, Leicester, United Kingdom

**Keywords:** *FRMD7*, Infantile nystagmus, X-linked inheritance, Penetrance, Genetic counseling

## Abstract

**Purpose:**

To determine whether mutation class (truncating versus nontruncating) modifies clinical penetrance in heterozygous female carriers of pathogenic *FRMD7* variants.

**Design:**

Retrospective multicohort pedigree analysis with cluster-aware statistical modeling.

**Subjects:**

There are 560 heterozygous female carriers from 126 pedigrees with pathogenic or likely pathogenic *FRMD7* variants: 100 pedigrees (477 carriers) from 40 published reports identified by systematic literature search and 26 pedigrees (83 carriers) from 2 UK institutional clinical genetics cohorts.

**Methods:**

Heterozygous female carriers were classified as affected or unaffected for infantile nystagmus based on clinical examination, and variants were stratified into truncating and nontruncating classes. Penetrance was compared between variant classes using generalized estimating equations with a logistic link and pedigree as the cluster variable. Sensitivity analyses comprised a mixed-effects logistic regression with a pedigree-level random intercept, together with leave-one-pedigree-out and leave-one-publication-out influence diagnostics.

**Main Outcome Measures:**

Clinical penetrance of infantile nystagmus, defined as the proportion of heterozygous female carriers meeting clinical criteria, compared between mutation classes.

**Results:**

Pooled penetrance was 30.1% (74/246; Wilson 95% confidence interval [CI] 24.7%–36.1%) for truncating variants and 49.7% (156/314; Wilson 95% CI 44.2%–55.2%) for nontruncating variants. Cluster-aware analysis confirmed higher odds of clinical manifestation for nontruncating than truncating variants (odds ratio [OR] 2.12, 95% CI 1.32–3.40, *P* = 0.002). The result was robust to leave-one-pedigree-out (OR range 2.02–2.28) and leave-one-publication-out (OR range 1.84–2.28) sensitivity analyses and was directionally concordant in literature and institutional cohorts (interaction *P* = 0.49).

**Conclusions:**

Mutation class is a robust modifier of clinical penetrance in heterozygous female carriers of pathogenic *FRMD7* variants. These variant-class specific estimates may refine genetic counseling in *FRMD7*-associated infantile nystagmus beyond the conventional single penetrance figure.

**Financial Disclosure(s):**

The authors have no proprietary or commercial interest in any materials discussed in this article.

Infantile nystagmus is an ocular disorder characterized by involuntary, rhythmic, and persistent oscillation of both eyes that usually appears within the first few months of life, with an estimated population prevalence of approximately 6.1 to 14 per 10 000 live births.^1^^,^^2^ Although infantile nystagmus is a heterogeneous group of disorders, most familial cases arise from pathogenic variants in the FERM domain–containing 7 gene (*FRMD7*) and are referred to as *FRMD7*-associated infantile nystagmus (FIN). *FRMD7*-associated infantile nystagmus typically presents within the first 6 months of life with horizontal conjugate oscillations, a reversed horizontal optokinetic response (OKR) alongside a preserved vertical OKR, and largely normal foveal architecture and electrophysiology.^3^, ^4^, ^5^, ^6^

*FRMD7*-associated infantile nystagmus shows a near-complete penetrance in hemizygous males but variable, incomplete penetrance in heterozygous female carriers, with reported estimates ranging from approximately 30% to 50% across individual family series.^3^^,^^4^ In addition to clinical nystagmus, subnormal OKRs have been observed in obligate carriers without overt nystagmus, suggesting a subclinical manifestation of the underlying phenotype.^3^ The mechanisms underlying this variable expressivity remain incompletely understood. One proposed explanation is skewed X-chromosome inactivation (XCI), in which prenatal selection favoring cells that have silenced the mutant allele leaves carrier females with a predominantly wild-type expression profile in the affected tissues.^7^ However, direct evidence for this mechanism in FIN is conflicting,^8^^,^^9^ which likely reflects the limitations of blood-based XCI assays for a gene principally expressed in developing brainstem and retinal tissue.^4^^,^^9^

Alongside XCI, the pathogenic variant itself may modify clinical expression.^10^ Functional studies of 2 *FRMD7* frameshift variants demonstrated that the mutant transcripts evade nonsense-mediated decay, but the resulting truncated proteins are subsequently cleared via the proteasomal pathway,^10^ consistent with these variants acting predominantly through reduced effective *FRMD7* dosage. Nontruncating variants, by contrast, may act through dominant-negative mechanisms. For example, the p.(Cys271Tyr) missense variant results in FRMD7 accumulation within the nucleus and inhibits wild-type–mediated neurite outgrowth, indicating that the abnormal protein interferes with residual wild-type function.^11^ These observations suggest that variant class may itself modulate penetrance in female carriers. A truncating variant producing simple haploinsufficiency could be compensated by preferential expression of the wild-type allele. By contrast, a nontruncating variant exerting dominant-negative effects could disrupt function even where the wild-type allele is predominantly expressed.

In this study, we assembled a multicohort data set combining 40 published *FRMD7* pedigrees with 2 UK institutional cohorts to test whether truncating versus nontruncating variants confer differential penetrance in heterozygous female carriers. We hypothesized, based on the molecular evidence summarized earlier, that nontruncating variants would be associated with higher penetrance than truncating variants.

## Methods

### Cohort Assembly

We assembled *FRMD7* pedigrees with confirmed pathogenic or likely pathogenic variants ascertained between August 2023 and September 2025 from a systematic literature search and 2 UK institutional cohorts. Literature pedigrees were identified through a systematic search of PubMed using terms combining *FRMD7* with "carrier," "heterozygote," "pedigree," "penetrance," "X-linked," and "infantile nystagmus," restricted to studies published in English between January 1990 and September 2025. Institutional pedigrees were drawn from 2 UK clinical genetics cohorts (the Leicester Ulverscroft Eye Unit and Moorfields Eye Hospital), comprising patients enrolled in research databases with appropriate ethical approval and written informed consent. The study adhered to the tenets of the Declaration of Helsinki. Ethical approval was granted by the East Midlands–Leicester Central Research Ethics Committee (REC references 20/EM/0040, 10/H0406/74, and 12/EM/0261).

Because the same family may appear in >1 publication, we undertook a structured deduplication step before inclusion. Each candidate pedigree was characterized by (1) the FRMD7 variant at cDNA and protein level in Human Genome Variation Society nomenclature, (2) the reporting centre and country, (3) author overlap across publications, (4) the number and sex of reported carriers and affected individuals, and (5) pedigree structure where a figure was available. Publications sharing one or more authors and reporting the same variant were compared directly, and where the same family could be identified across reports the most complete account was retained and the duplicate removed. A detailed breakdown of pedigrees from all sources can be found in Supplementary Table 1 (available at www.ophthalmologyscience.org). All identified pedigrees were subsequently screened for inclusion based on predefined variant-level and clinical criteria.

#### Variant-Level Inclusion

Pedigrees were included only where the segregating *FRMD7* variant was classifiable as pathogenic or likely pathogenic according to the American College of Medical Genetics and Genomics/Association for Molecular Pathology (ACMG/AMP) 2015 criteria,^12^ supported by ACMG/AMP classification. Where the source publication provided no explicit classification, or predated the 2015 ACMG/AMP framework, the variant was reclassified independently for this study using the 2015 ACMG/AMP criteria, drawing on gnomAD allele frequencies, in silico predictors, and available segregation and functional evidence. Only variants reaching pathogenic or likely pathogenic when considering all available criteria were retained and variants of uncertain significance, benign, and likely benign variants were excluded. Each variant was further classified for the primary analysis into 1 of 2 mutation classes. Truncating variants comprised nonsense, frameshift and canonical splice-site variants, together with whole-exon deletions and start-loss variants. Nontruncating variants comprised missense changes, in-frame insertions and deletions, and noncanonical splice-site variants. This binary stratification reflects the expected mechanistic distinction between loss-of-function (predominantly through nonsense-mediated decay) and variants likely to produce altered or partially functional protein and provides a clinically tractable framework relevant to genetic counseling.

#### Clinical Inclusion

Pedigrees were retained only where heterozygous female carriers could be definitively classified as either affected or unaffected. Affected status required clinical documentation of infantile nystagmus consistent with FIN (horizontal conjugate oscillation with onset in infancy) on ophthalmic examination. Electrophysiology and ocular motility recording (eye movement recording, OKR testing) were not required for classification and were not uniformly available across sources, particularly in older literature reports. Where reported however, they were used to support the diagnosis. Heterozygous females not meeting these criteria were classified as unaffected. Pedigrees in which carrier status of any female could not be definitively established were excluded.

### Statistical Analysis

#### Primary Analysis

The primary outcome was clinical penetrance, defined as the proportion of affected heterozygous female carriers within each variant class. Pooled penetrance estimates were calculated together with 2-sided 95% Wilson confidence intervals (CIs). Because heterozygous female carriers within a single pedigree share genetic background, XCI patterns inherited from common ancestors, environmental exposures, and ascertainment, they cannot be treated as independent observations in formal hypothesis testing. We therefore compared penetrance between truncating and nontruncating variants using generalized estimating equations (GEEs)^13^ with logistic link, exchangeable working correlation structure, and pedigree as the cluster variable. Odds ratios (ORs) with 95% CIs were derived by exponentiation of the log-odds coefficient and its bounds (estimate ± 1.96× standard error). In addition, a nonparametric Mann–Whitney comparison of per-pedigree penetrance was performed among multicarrier pedigrees, in which each pedigree contributed a single observation. Pedigrees contributing only a single heterozygous female carrier were excluded from this analysis.

#### Sensitivity Analysis

To rule out the influence of individual pedigrees or studies on penetrance estimates we conducted 3 sensitivity analyses. First, a mixed-effects logistic regression was fitted with a random intercept per pedigree, providing a subject-specific (conditional) OR as a complementary estimate to the population-averaged GEE OR. Second, the cluster-robust GEE was refitted iteratively after removing 1 pedigree at a time (leave-one-pedigree-out), and after sequentially excluding all pedigrees from each contributing publication (leave-one-PMID-out). These were treated as descriptive influence diagnostics rather than independent hypothesis tests. Third, we assessed whether the data source confounded or modified the variant-class effect, given that literature pedigrees may be ascertained based on informativeness for publication, whereas institutional cohorts are recruited though clinical referral. To test for confounding, we fitted an additive source-adjusted GEE (affected ∼ variant class + source). To test for effect modification, we fitted an interaction GEE (affected ∼ variant class × source). Both models used cluster-robust standard errors. All analyses were conducted in R version 4.5.1 (R Foundation for Statistical Computing), using geepack^14^ (1.3.13) for GEE models, lme4^15^ (1.1-38) for mixed-effects models, and binom (https://cran.r-project.org/web/packages/binom/index.html) (1.1) for Wilson CIs. A 2-sided significance threshold of α = 0.05 was applied.

## Results

### Study Cohort

We assembled 126 *FRMD7* pedigrees comprising 560 heterozygous female carriers, of whom 230 (41.1%) met clinical criteria for infantile nystagmus. One hundred pedigrees (477 carriers) were derived from 40 published reports identified through systematic literature search; 26 pedigrees (83 carriers) were ascertained from the 2 UK institutional cohorts (Supplementary Table 1). The cohort summary stratified by data source and variant class is presented in Table 1.

**Table 1:** *FRMD7* Heterozygous Female Carrier Cohort by Data Source and Variant Class

Source	Variant Class	Pedigrees	Carriers	Affected
Overall	All variants	126	560	230
Overall	Truncating	59	246	74
Overall	Nontruncating	67	314	156
Institutional	Truncating	12	37	13
Institutional	Nontruncating	14	46	20
Literature	Truncating	47	209	61
Literature	Nontruncating	53	268	136

### Penetrance by Variant Class

**Figure 1:**
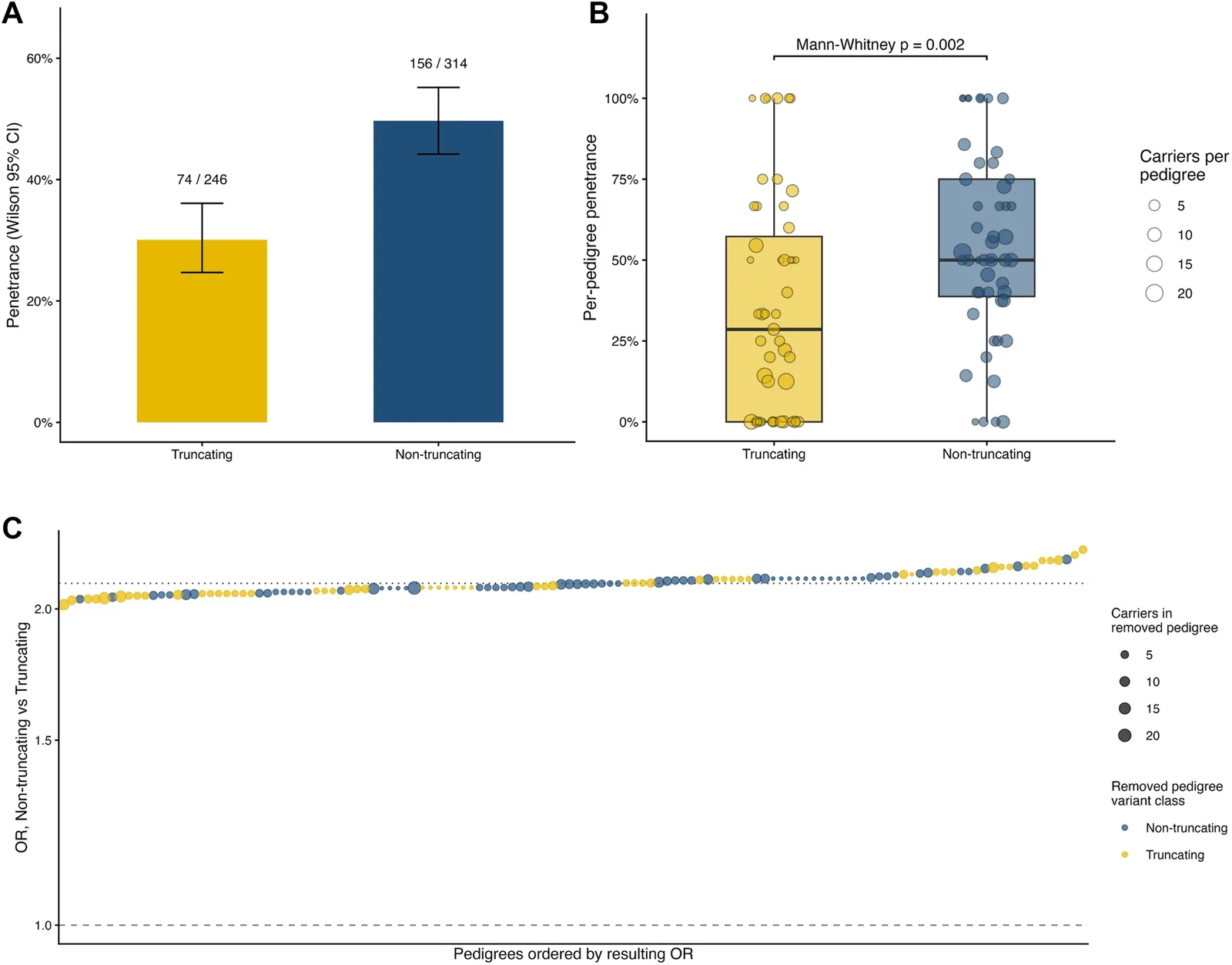
Variant class is a robust modifier of clinical penetrance in heterozygous female *FRMD7* carriers. (**A**) Pooled clinical penetrance by variant class with 2-sided 95% Wilson CIs. Counts of affected over total carriers are annotated above each bar. (**B**) Distribution of per-pedigree clinical penetrance among multicarrier pedigrees (n = 102 with ≥2 heterozygous female carriers); each point represents 1 pedigree, and point size is proportional to the number of heterozygous female carriers in that pedigree. (**C**) Leave-one-pedigree-out sensitivity analysis. Each point represents the variant-class OR from the primary GEE model after refitting with 1 pedigree excluded. Point color indicates the variant class of the excluded pedigree (yellow = truncating; dark blue = nontruncating); point size is proportional to the number of heterozygous female carriers in the excluded pedigree. CI = confidence interval; GEE = generalized estimating equation; OR = odds ratio.

Pooled clinical penetrance among heterozygous female *FRMD7* carriers was 30.1% (74/246; Wilson 95% CI 24.7%–36.1%) for truncating variants and 49.7% (156/314; Wilson 95% CI 44.2%–55.2%) for nontruncating variants (Fig 1A). After accounting for within-pedigree clustering using a generalized estimating equation, heterozygous females carrying nontruncating *FRMD7* pathogenic variants had higher odds of clinical manifestation than carriers of truncating variants (OR 2.12, 95% CI 1.32–3.40, *P* = 0.002). A mixed-effects logistic regression with a random intercept per-pedigree gave a concordant effect direction with a larger conditional OR (OR 2.45, 95% CI 1.44–4.20, *P* = 0.001), as expected because subject-specific estimators are typically larger in magnitude than population-averaged estimators when between-cluster variance is non-negligible. A complementary nonparametric Mann–Whitney comparison among multicarrier pedigrees (n = 102), in which each pedigree contributed a single observation and within-pedigree clustering was therefore eliminated by construction, supported the same conclusion. Median per-pedigree penetrance was 0.50 (interquartile range 0.39–0.75) for nontruncating variants compared with 0.29 (interquartile range 0.00–0.57) for truncating variants (*P* = 0.002; Fig 1B).

### Sensitivity Analyses

To assess whether the primary result was driven by any individual family, we refitted the GEE model 126 times, each time excluding 1 pedigree. The variant-class OR remained between 2.02 and 2.28 across all iterations, with the 95% CI excluding the null in every case (Fig 1C; Supplementary Table 2, available at www.ophthalmologyscience.org). To assess whether any single publication's ascertainment was influential, we refitted the model 40 times, excluding each publication's pedigrees in turn; the OR remained between 1.84 and 2.28 with the CI excluding 1 throughout (Supplementary Table 3, available at www.ophthalmologyscience.org).

A pooled GEE adjusted for data source (i.e., literature or institutional) produced an essentially unchanged variant-class OR (OR 2.11, 95% CI 1.31–3.40, *P* = 0.002), with no evidence of a main effect of data source on carrier affected status (*P* = 0.92), indicating that source did not act as a confounder of the variant-class effect. A formal test for effect modification using a variant-class X source interaction term was not statistically significant (OR 1.64, 95% CI 0.40–6.65, interaction *P* = 0.49). Together, these analyses argue against the variant-class effect being an artefact of either individual pedigree influence or differential ascertainment between literature and institutional sources.

## Discussion

In a multicohort analysis of 126 *FRMD7* pedigrees comprising 560 heterozygous female carriers, we demonstrate that mutation class is a robust modifier of clinical penetrance for infantile nystagmus. Heterozygous females carrying nontruncating *FRMD7* variants had approximately twice the odds of clinical manifestation compared with carriers of truncating variants (OR 2.12, 95% CI 1.32–3.40), corresponding to absolute penetrance estimates of approximately 50% and 30%, respectively. The result is robust to influence diagnostics at both the pedigree and publication level, is directionally concordant across literature and institutional cohorts, and is supported by a nonparametric pedigree-level analysis that eliminates within-pedigree clustering by construction. To our knowledge, this is the first study to provide variant-class–stratified penetrance estimates for *FRMD7* using cluster-aware methods.

Our pooled nontruncating penetrance estimate of 50% closely matches the 30%–50% figure that has historically been cited in clinical guidelines and reviews of FIN,^3^^,^^4^ but the corresponding 30% estimate for truncating variants has not previously been reported as a separable quantity. The clinical implication is direct, because variant-specific estimates can be incorporated into genetic counselling using a binary truncating-versus-nontruncating distinction that requires no mechanistic interpretation at the point of variant reporting. For heterozygous females identified through cascade testing, the conventional approximately 50% penetrance estimate refines to roughly 1 in 2 for nontruncating carriers and 1 in 3 for truncating carriers. These are cohort-level averages conditional on variant class, and are best considered alongside family-specific factors, the pattern of X-chromosome inactivation in relevant tissues, ancestry, the specific variant, and the ascertainment context in which a carrier was identified, to support more precise genetic counselling in FIN.

Several biological mechanisms could plausibly explain the higher penetrance observed for nontruncating variants. Functional studies of 2 *FRMD7* frameshift variants showed that mutant transcripts evade nonsense-mediated decay, but the resulting truncated proteins are cleared via the proteasomal pathway,^10^ consistent with reduced effective FRMD7 dosage. By contrast, the p.(Cys271Tyr) missense variant mislocalizes to the nucleus and inhibits wild-type FRMD7-mediated neurite outgrowth,^11^ a dominant-negative mechanism in which the variant protein interferes with residual wild-type function. This molecular asymmetry, predominantly loss-of-function for truncating variants, potentially dominant-negative for nontruncating variants, provides a mechanistic basis for the differential penetrance observed clinically, because dominant-negative interference would be expected to manifest in any cell expressing the variant allele rather than depending solely on the proportion of cells in which the variant X is active.

This framework also offers an explanation for variable penetrance among carriers of the same variant class. *FRMD7* is dynamically expressed during human embryonic and foetal retinal development^4^^,^^6^ and is required for the establishment of asymmetric inhibitory inputs from starburst amacrine cells to direction-selective retinal ganglion cells.^16^ Cells expressing severely loss-of-function (truncating) variants may be at a developmental disadvantage during the critical window of retinal and oculomotor circuit formation, and could be selectively eliminated or outcompeted by cells in which the wild-type allele is on the active X chromosome. Such prenatal cell-lineage selection would phenocopy lineage-specific X-inactivation skewing in the affected tissues without producing a detectable signal in peripheral blood, potentially reconciling the conflicting reports of skewed and unskewed X-inactivation in FIN carriers.^8^^,^^9^ Direct functional comparisons of truncating and nontruncating *FRMD7* variants in patient-derived neuronal models, combined with cell-type–specific X-inactivation analysis in oculomotor tissues, are needed to test these hypotheses formally.

We acknowledge several limitations of this study. First, the cohort is heavily weighted toward literature pedigrees (100 of 126), which carry an inherent risk of ascertainment bias toward symptomatic families. Our sensitivity analyses confirmed that the variant-class effect was not driven by individual pedigrees, single publications, or differential ascertainment. Nonetheless, the institutional cohort (26 pedigrees, 83 carriers) is not independently powered to confirm the effect, and establishes directional concordance with the literature-derived estimate rather than validating it as a standalone replication. A degree of upward inflation in the literature-derived estimate therefore cannot be excluded. Prospectively ascertained cohorts will be required to confirm the effect and refine within-source estimates.

Second, the dichotomization of variants into truncating and nontruncating classes inevitably aggregates over heterogeneous molecular consequences. The binary classification aggregates over heterogeneous molecular consequences, and several classes sit less comfortably at the boundary than clear nonsense or missense variants, including start-loss, whole-exon deletion, noncanonical splice-site, and in-frame variants. Future studies with larger cohorts could resolve penetrance at the level of individual variants or finer mechanistic subclasses, which our sample size did not permit. Within the truncating class, frameshift variants escaping nonsense-mediated decay^10^ may behave differently from those undergoing decay; within the nontruncating class, dominant-negative missense variants may differ from those acting purely through reduced protein function. We chose the binary classification because it provides a clinically tractable framework relevant to genetic counseling and because finer subclassification would severely reduce statistical power.

Third, ascertainment of unaffected carriers requires reliable testing of asymptomatic family members. We cannot exclude the possibility that mildly affected carriers with subclinical eye movement abnormalities not detected on routine examination were classified as unaffected, particularly in older literature reports that did not employ ocular motility recording, OKR testing, or electrophysiology systematically.^3^ Ascertainment of the unaffected state was therefore not uniform across sources, and mild or subclinical carriers may have been misclassified as unaffected more often in reports relying on routine examination alone. This would tend to bias absolute penetrance downward but would not be expected to distort the relative comparison between variant classes.

In summary, we provide robust, variant-specific clinical penetrance estimates for heterozygous females carrying pathogenic *FRMD7* variants. The approximately 20 percentage-point absolute difference in penetrance between truncating and nontruncating variants is methodologically robust to cluster-aware reanalysis and directly applicable to genetic counseling. We suggest that the truncating-versus-nontruncating distinction merits consideration in *FRMD7* carrier counseling as a cohort-level refinement to the conventional 50% penetrance estimate, pending validation in prospectively ascertained cohorts. Functional studies dissecting the differential cellular consequences of truncating and nontruncating *FRMD7* variants in human neuronal models, together with cell-type–specific X-inactivation analysis, will be needed to establish the molecular basis of this clinical observation.
