# Supplementary material for: Nontruncating FRMD7 Variants Are More Penetrant than Truncating Variants in Heterozygous Female Carriers of Infantile Nystagmus
Source: Ophthalmol Sci. 2026 Aug 14;6(10):101363. doi: 10.1016/j.xops.2026.101363 (PMC13589131; doi:10.1016/j.xops.2026.101363)
Supplement: Supplementary file 1 [file mmc1.pdf]

Supplementary Table 1: Overview of FRMD7 pedigrees from literature and institutional searches

Gene	Source_Type	PMID	Family_Label	Ethnicity	Variant_Location	cDNA	Protein_Change	Variant_Class	Affected_Females	Unaffected_Female_Carriers	Total_Female_Carriers	Pedigree ID
FRMD7	Literature	26268155	F1	Korean	Exon 1	c.1A>G	p.M1V	Truncating	2	0	2	F_1
FRMD7	Literature	26268155	F2	Korean	Exon 1	c.1A>G	p.M1V	Truncating	1	1	2	F_2
FRMD7	Literature	30618027	F1	Chinese	Exon 1	c.47T>C	p.F16S	Non-truncating	3	4	7	F_3
FRMD7	Literature	21303855	F8	England	Exon 1	c.47T>C	p.F16S	Non-truncating	2	1	3	F_4
FRMD7	Institutional	NA	F109	Caucasian	Exon 1	c.47T>C	p.F16S	Non-truncating	2	1	3	F_5
FRMD7	Literature	25678693	F1	Belgian	Exon 2	c.70G>A	p.G24R	Non-truncating	0	1	1	F_6
FRMD7	Literature	30942644	F1	-	Exon 2	c.70G>A	p.G24R	Non-truncating	2	3	5	F_7
FRMD7	Literature	17013395	F15	Ireland	Exon 2	c.70G>A	p.G24R	Non-truncating	3	3	6	F_8
FRMD7	Literature	21303855	F2	Ireland	Exon 2	c.70G>A	p.G24R	Non-truncating	3	5	8	F_9
FRMD7	Literature	18431453	F4	Chinese	Exon 2	c.70G>T	p.G24W	Non-truncating	6	1	7	F_10
FRMD7	Literature	37423457	F6	-	Exon 4	c.234G>A	p.M78I	Non-truncating	4	1	5	F_11
FRMD7	Literature	34132631	F5	-	Exon 4	c.234G>A	p.M78I	Non-truncating	1	0	1	F_12
FRMD7	Literature	33732697	F4	Chinese	Exon 4	c.284G>A	p.R95K	Non-truncating	1	0	1	F_13
FRMD7	Institutional	NA	F126	Caucasian	Exon 5	c.290T>C	p.L97P	Non-truncating	1	0	1	F_14
FRMD7	Institutional	NA	F116	Caucasian	Exon 6	c.425T>C	p.L142P	Non-truncating	1	6	7	F_15
FRMD7	Literature	17013395	F16	Ireland	Exon 6	c.425T>G	p.L142R	Non-truncating	6	2	8	F_16
FRMD7	Literature	25916882	F1	North Indian	Exon 7	c.556A>G	p.M186V	Non-truncating	0	3	3	F_17
FRMD7	Literature	30025138	F5	Korean	Exon 7	c.575A>C	p.H192P	Non-truncating	1	3	4	F_18
FRMD7	Literature	30025138	F6	Korean	Exon 7	c.575A>C	p.H192P	Non-truncating	0	1	1	F_19
FRMD7	Literature	32446246	F2	Chinese	Exon 7	c.580G>A	p.A194T	Non-truncating	2	0	2	F_20
FRMD7	Literature	21365021	F2	Chinese	Exon 7	c.623A>G	p.H208R	Non-truncating	0	8	8	F_21
FRMD7	Literature	23946638	F1	Chinese	Exon 7	c.635T>C	p.L212P	Non-truncating	3	2	5	F_22
FRMD7	Literature	34132631	F6	-	Exon 8	c.661A>C	p.N221H	Non-truncating	3	1	4	F_23

FRMD7	Institutional	NA	F136	Caucasian	Exon 8	c.670A>G	p.N224D	Non-truncating	1	7	8	F_24
FRMD7	Institutional	NA	F119	Caucasian	Exon 8	c.673T>C	p.W225R	Non-truncating	2	4	6	F_25
FRMD7	Literature	21303855	F7	England	Exon 8	c.676G>A	p.A226T	Non-truncating	2	0	2	F_26
FRMD7	Literature	17962394	F1	Turkish	Exon 8	c.686C>G	p.R229G	Non-truncating	4	6	10	F_27
FRMD7	Literature	17013395	F3	Ireland-Germany	Exon 8	c.691T>G	p.L231V	Non-truncating	4	4	8	F_28
FRMD7	Literature	21303855	F1	Ireland & England	Exon 8	c.691T>G	p.L231V	Non-truncating	5	5	10	F_29
FRMD7	Institutional	NA	F105	Caucasian	Exon 8	c.713T>C	p.F238S	Non-truncating	0	3	3	F_30
FRMD7	Literature	24169426	F1	-	Exon 8	c.719T>C	p.I240T	Non-truncating	4	4	8	F_31
FRMD7	Literature	35705619	F26	Chinese	Exon 9	c.773T>C	p.M258T	Non-truncating	2	6	8	F_32
FRMD7	Literature	35705619	F27	Chinese	Exon 9	c.773T>C	p.M258T	Non-truncating	5	6	11	F_33
FRMD7	Literature	24434814	F1	-	Exon 9	c.780C>A	p.S260R	Non-truncating	4	1	5	F_34
FRMD7	Literature	33732697	F3	Chinese	Exon 9	c.781C>G	p.R261G	Non-truncating	0	1	1	F_35
FRMD7	Literature	27036142	F2	Chinese	Exon 9	c.781C>G	p.R261G	Non-truncating	0	2	2	F_36
FRMD7	Literature	23733424	F1	Chinese	Exon 9	c.781G>C	p.R261G	Non-truncating	3	3	6	F_37
FRMD7	Literature	18431453	F5	Chinese	Exon 9	c.782G>A	p.R261Q	Non-truncating	2	3	5	F_38
FRMD7	Literature	18431453	F6	Chinese	Exon 9	c.782G>A	p.R261Q	Non-truncating	2	2	4	F_39
FRMD7	Literature	33732697	F1	Chinese	Exon 9	c.782G>A	p.R261Q	Non-truncating	0	1	1	F_40
FRMD7	Literature	33732697	F2	Chinese	Exon 9	c.782G>A	p.R261Q	Non-truncating	0	1	1	F_41
FRMD7	Institutional	NA	F122	Caucasian	Exon 9	c.782G>A	p.R261Q	Non-truncating	5	1	6	F_42
FRMD7	Literature	17013395	F6	England	Exon 9	c.796G>C	p.A266P	Non-truncating	3	0	3	F_43
FRMD7	Literature	21303855	F4	England	Exon 9	c.796G>C	p.A266P	Non-truncating	2	1	3	F_44
FRMD7	Literature	28378818	F1	-	Exon 9	c.796G>C	p.(A266P)	Non-truncating	1	1	2	F_45
FRMD7	Literature	25678693	F8	Belgian	Exon 9	c.801C>A	p.F267L	Non-truncating	1	0	1	F_46
FRMD7	Literature	30576400	F1	Chinese	Exon 9	c.805A>C	p.K269Q	Non-truncating	1	4	5	F_47
FRMD7	Literature	17013395	F11	Scotland	Exon 9	c.812G>A	p.C271Y	Non-truncating	8	3	11	F_48

FRMD7	Literature	21303855	F3	England	Exon 9	c.812G>A	p.C271Y	Non-truncating	5	0	5	F_49
FRMD7	Literature	18431453	F1	Chinese	Exon 9	c.812G>T	p.C271F	Non-truncating	8	6	14	F_50
FRMD7	Literature	18246032	F1	Chinese	Exon 9	c.812G>T	p.C271F	Non-truncating	11	10	21	F_51
FRMD7	Literature	30025138	F4	Korean	Exon 9	c.875T>C	p.L292P	Non-truncating	2	1	3	F_52
FRMD7	Literature	25678693	F9	Belgian	Exon 9	c.875T>C	p.L292P	Non-truncating	0	1	1	F_53
FRMD7	Literature	27081518	F1	Japanese	Exon 9	c.875T>C	p.L292P	Non-truncating	0	1	1	F_54
FRMD7	Literature	34132631	F7	-	Exon 9	c.875T>C	p.L292P	Non-truncating	1	3	4	F_55
FRMD7	Literature	25678693	F3	Belgian	Exon 9	c.886G>C	p.G296R	Non-truncating	0	1	1	F_56
FRMD7	Institutional	NA	F133	Caucasian	Exon 9	c.886G>C	p.G296R	Non-truncating	3	0	3	F_57
FRMD7	Literature	30015830	F1	Chinese	Exon 9	c.886G>T	p.G296C	Non-truncating	0	1	1	F_58
FRMD7	Literature	17013395	F12	England	Exon 9	c.902A>G	p.Y301C	Non-truncating	2	3	5	F_59
FRMD7	Literature	22490987	F1	South Indian	Exon 10	c.917 A>G	p.Q305R	Non-truncating	3	5	8	F_60
FRMD7	Literature	32446246	F3	Chinese	Exon 10	c.973A>G	p.R325G	Non-truncating	5	4	9	F_61
FRMD7	Institutional	NA	F106	Caucasian	Exon 11	c.983A>G	p.Y328C	Non-truncating	2	0	2	F_62
FRMD7	Literature	21303855	F10	Romania	Exon 11	c.1019C>T	p.S340L	Non-truncating	2	0	2	F_63
FRMD7	Literature	37423457	F5	-	Exon 12	c.1109G>A	p.H370R	Non-truncating	4	3	7	F_64
FRMD7	Institutional	NA	F137	Caucasian	Exon 2	c.154G>T	p.G52*	Truncating	0	3	3	F_65
FRMD7	Literature	36833273	F1	Pakistani	Exon 6	c.443T>A	p.L148*	Truncating	1	1	2	F_66
FRMD7	Literature	17013395	F2	Italy-Germany	Exon 7	c.601C>T	p.Q201*	Truncating	2	4	6	F_67
FRMD7	Literature	18431453	F7	Chinese	Exon 10	c.910C>T	p.R304*	Truncating	1	4	5	F_68
FRMD7	Literature	25678693	F2	Belgian	Exon 10	c.910C>T	p.R304*	Truncating	1	1	2	F_69
FRMD7	Literature	17013395	F10	England	Exon 11	c.1003C>T	p.R335*	Truncating	1	1	2	F_70
FRMD7	Literature	24513357	F1	Chinese	Exon 11	c.1003C>T	p.R335*	Truncating	0	4	4	F_71
FRMD7	Literature	21303855	F5	England	Exon 11	c.1003C>T	p.R335*	Truncating	0	2	2	F_72
FRMD7	Literature	21303855	F6	India	Exon 11	c.1003C>T	p.R335*	Truncating	0	3	3	F_73

FRMD7	Institutional	NA	F104	South Asian	Exon 11	c.1003C>T	p.R335*	Truncating	0	1	1	F_74
FRMD7	Literature	27036142	F1	Chinese	Exon 12	c.1090C>T	p.Q364*	Truncating	0	6	6	F_75
FRMD7	Literature	24434814	F2	-	Exon 12	c.1458G>T	p.Q487*	Truncating	0	3	3	F_76
FRMD7	Literature	20450309	F1	-	Exons 2-4	c.58_284del	p.(Q20Vfs*20)	Truncating	2	7	9	F_77
FRMD7	Literature	25678693	F4	Belgian	Exon 8	c.660delC	p.N221Ifs*11	Truncating	0	3	3	F_78
FRMD7	Literature	18431453	F2	Chinese	Exon 8	c.689_690delAG	p.K230Ifs*4	Truncating	6	5	11	F_79
FRMD7	Literature	30616528	F1	Chinese	Exon 9	c.823_829delCATGCTT	p.H275Sfs*42	Truncating	5	2	7	F_80
FRMD7	Literature	17846367	F1	-	Exon 9	c.880_881insA	p.S294Kfs*302	Truncating	2	12	14	F_81
FRMD7	Literature	17013395	F14	Austria	Exon 9	c.887delG	p.G296Vfs*23	Truncating	3	0	3	F_82
FRMD7	Literature	23733424	F2	Chinese	Exon 11	c.980_983delATTA	p.H327Pfs*353	Truncating	0	3	3	F_83
FRMD7	Literature	32446246	F1	Chinese	Exon 11	c.998dupA	p.H333Qfs*2	Truncating	0	13	13	F_84
FRMD7	Literature	28378818	F3	-	Exon 12	c.1262delC	p.(P421Lfs*23)	Truncating	3	3	6	F_85
FRMD7	Literature	31495972	F1	Chinese	Exon 12	c.1419_1422dupAACT	p.Y475Nfs*39	Truncating	2	5	7	F_86
FRMD7	Literature	22065930	F1	Chinese	Exon 12	c.1486_1489delTTTT	p.F497Mfs*26	Truncating	0	7	7	F_87
FRMD7	Literature	37423457	F2	-	Exon 12	c.1492_1493insT	p.Y498Lfs*14	Truncating	0	4	4	F_88
FRMD7	Literature	37545716	F1	-	Exon 12	c.1492dupT	p.Y498Lfs*15	Truncating	1	4	5	F_89
FRMD7	Literature	24434814	F3	-	Exon 12	c.1645delG	p.V549Yfs*554	Truncating	0	4	4	F_90
FRMD7	Literature	25678693	F7	Belgian	Exon 12	c.2036delT	p.L679Rfs*8	Truncating	0	1	1	F_91
FRMD7	Literature	21303855	F9	England	Intron 1	c.58-1G>A	-	Truncating	0	1	1	F_92

FRMD7	Literature	17013395	F7	England	Intron 2	c.162+5G>A	-	Truncating	1	2	3	F_93
FRMD7	Institutional	NA	F110	Caucasian	Intron 2	c.162+5G>A	-	Truncating	1	0	1	F_94
FRMD7	Literature	30025138	F3	Korean	Intron 2	c.162+6T>C	-	Truncating	1	1	2	F_95
FRMD7	Literature	22262942	F1	Chinese	Intron 2	c.163–1G>T	-	Truncating	5	0	5	F_96
FRMD7	Literature	17013395	F4	England	Intron 3	c.205+2T>G	-	Truncating	2	3	5	F_97
FRMD7	Institutional	NA	F121	Caucasian	Intron 3	c.206-5T>A	-	Truncating	2	0	2	F_98
FRMD7	Literature	33732697	F5	Chinese	Intron 4	c.284+10T>G	-	Truncating	3	2	5	F_99
FRMD7	Literature	17013395	F1	England	Intron 4	c.284+1G>A	-	Truncating	2	14	16	F_100
FRMD7	Literature	35762937	F2	-	Intron 4	c.284+63T>A	-	Truncating	3	0	3	F_101
FRMD7	Literature	35762937	F3	-	Intron 4	c.284+63T>A	-	Truncating	0	1	1	F_102
FRMD7	Literature	35762937	F1	-	Intron 4	c.285-12A>G	-	Truncating	0	3	3	F_103
FRMD7	Literature	37423457	F12	-	Intron 5	c.382+1G>C	-	Truncating	1	2	3	F_104
FRMD7	Institutional	NA	F129	Caucasian	Intron 5	c.382+7_382+8insT	-	Truncating	2	1	3	F_105
FRMD7	Literature	35762937	F4	-	Intron 5	c.383-1368A>G	-	Truncating	2	1	3	F_106
FRMD7	Literature	25678693	F10	Belgian	Intron 6	c.497+5G>A	-	Truncating	0	2	2	F_107
FRMD7	Literature	30890130	F1	Chinese	Intron 6	c.498-3C>T	-	Truncating	0	3	3	F_108
FRMD7	Literature	17013395	F13	Madagascar	Intron 7	c.645+1G>C	-	Truncating	3	1	4	F_109
FRMD7	Literature	17013395	F9	Germany	Intron 11	c.1050+1G>C	-	Truncating	1	3	4	F_110
FRMD7	Literature	21746984	F1	-	Intron 11	c.1050+5G>A	-	Truncating	1	3	4	F_111
FRMD7	Literature	17013395	F5	England	Exon 4	c.252G>A	p.V84V	Truncating	0	1	1	F_112

FRMD7	Institutional	NA	F31	-	Exon 1	c.41_43delAGA	p.K14del	Truncating	1	2	3	F_113
FRMD7	Literature	33531592	F3	-	Exons 2-12	c.58_2145del	-	Truncating	0	1	1	F_114
FRMD7	Literature	28378818	F7	-	Exons 2-12	c.58_2145del	-	Truncating	3	1	4	F_115
FRMD7	Institutional	NA	NA	NA	NA	c.206-5T>A	NA	Truncating	2	1	3	F_116
FRMD7	Institutional	NA	NA	NA	NA	c.1003C>T;	p.(Arg335*)	Truncating	4	0	4	F_117
FRMD7	Institutional	NA	NA	NA	NA	c.790T>G	p.Cys264Gly	Non-truncating	0	1	1	F_118
FRMD7	Institutional	NA	NA	NA	NA	c.875T>C;	p.(Leu292Pro)	Non-truncating	1	1	2	F_119
FRMD7	Institutional	NA	NA	NA	NA	c.383-1G>A	NA	Non-truncating	0	1	1	F_120
FRMD7	Institutional	NA	NA	NA	NA	c.796G>C	p.(Ala266Pro)	Non-truncating	0	1	1	F_121
FRMD7	Institutional	NA	NA	NA	NA	c.96C>A	p.(Cys32*)	Truncating	0	2	2	F_122
FRMD7	Institutional	NA	NA	NA	NA	c.590del	p.Gly197Valfs*35	Truncating	1	7	8	F_123
FRMD7	Institutional	NA	NA	NA	NA	c.1003C>T	p.(Arg335*)	Truncating	0	1	1	F_124
FRMD7	Institutional	NA	NA	NA	NA	c.675G>A	p.(Trp225*)	Truncating	0	6	6	F_125
FRMD7	Institutional	NA	NA	NA	NA	c.379C>A	p.(Gln127Lys)	Non-truncating	2	0	2	F_126
