# Supplementary material for: Nontruncating FRMD7 Variants Are More Penetrant than Truncating Variants in Heterozygous Female Carriers of Infantile Nystagmus
Source: Ophthalmol Sci. 2026 Aug 14;6(10):101363. doi: 10.1016/j.xops.2026.101363 (PMC13589131; doi:10.1016/j.xops.2026.101363)
Supplement: Supplementary file 2 [file mmc2.pdf]

Supplementary Table 2: Generalised estimating equation leave-one-pedigree-out results

Removed Pedigree ID	Number of Carriers Removed	Variant Class	OR	CI Lower	CI Higher	P
**F_1**	2	Truncating	2.192724847	1.36037009	3.53436341	0.00126613
**F_2**	2	Truncating	2.13476731	1.32185474	3.44760382	0.00192863
**F_3**	7	Non-truncating	2.125523363	1.31759668	3.42885621	0.0019986
**F_4**	3	Non-truncating	2.096111198	1.30140221	3.37611395	0.00233997
**F_5**	3	Non-truncating	2.096111198	1.30140221	3.37611395	0.00233997
**F_6**	1	Non-truncating	2.13719275	1.3286532	3.43776151	0.00173768
**F_7**	5	Non-truncating	2.128635187	1.32040379	3.43159252	0.00193031
**F_8**	6	Non-truncating	2.11263104	1.30976493	3.40764195	0.00216734
**F_9**	8	Non-truncating	2.137143735	1.32501937	3.44703138	0.00184654
**F_10**	7	Non-truncating	2.051910499	1.27717858	3.29659201	0.0029645
**F_11**	5	Non-truncating	2.069542575	1.28603102	3.33040682	0.00273236
**F_12**	1	Non-truncating	2.092782244	1.30102071	3.36638571	0.00232643
**F_13**	1	Non-truncating	2.092782244	1.30102071	3.36638571	0.00232643
**F_14**	1	Non-truncating	2.092782244	1.30102071	3.36638571	0.00232643
**F_15**	7	Non-truncating	2.184568655	1.35966588	3.50993598	0.00123808
**F_16**	8	Non-truncating	2.065818997	1.28313726	3.32591709	0.00282568
**F_17**	3	Non-truncating	2.170632348	1.3509372	3.48768604	0.00135895
**F_18**	4	Non-truncating	2.147740279	1.33383247	3.45829661	0.00165963
**F_19**	1	Non-truncating	2.13719275	1.3286532	3.43776151	0.00173768
**F_20**	2	Non-truncating	2.075609106	1.29082453	3.33752038	0.00258313
**F_21**	8	Non-truncating	2.228799286	1.39454124	3.56213651	0.00080774
**F_22**	5	Non-truncating	2.098086387	1.30146125	3.3823262	0.00235438

**F_23**	4	Non-truncating	2.081440535	1.29266447	3.35152299	0.00255934
**F_24**	8	Non-truncating	2.195246661	1.36758424	3.52381064	0.00112792
**F_25**	6	Non-truncating	2.141926443	1.32893229	3.4522819	0.00176182
**F_26**	2	Non-truncating	2.075609106	1.29082453	3.33752038	0.00258313
**F_27**	10	Non-truncating	2.132978812	1.32171018	3.44220593	0.00192024
**F_28**	8	Non-truncating	2.111552724	1.30839441	3.40773	0.00220775
**F_29**	10	Non-truncating	2.110303566	1.30706198	3.40716908	0.00224602
**F_30**	3	Non-truncating	2.170632348	1.3509372	3.48768604	0.00135895
**F_31**	8	Non-truncating	2.111552724	1.30839441	3.40773	0.00220775
**F_32**	8	Non-truncating	2.164899918	1.34461273	3.48560708	0.00148002
**F_33**	11	Non-truncating	2.120084275	1.31300854	3.42325064	0.00211219
**F_34**	5	Non-truncating	2.069542575	1.28603102	3.33040682	0.00273236
**F_35**	1	Non-truncating	2.13719275	1.3286532	3.43776151	0.00173768
**F_36**	2	Non-truncating	2.155330218	1.34042474	3.46565399	0.00152931
**F_37**	6	Non-truncating	2.11263104	1.30976493	3.40764195	0.00216734
**F_38**	5	Non-truncating	2.128635187	1.32040379	3.43159252	0.00193031
**F_39**	4	Non-truncating	2.113552165	1.3112555	3.4067371	0.00212214
**F_40**	1	Non-truncating	2.13719275	1.3286532	3.43776151	0.00173768
**F_41**	1	Non-truncating	2.13719275	1.3286532	3.43776151	0.00173768
**F_42**	6	Non-truncating	2.059842127	1.28098673	3.31225101	0.00286554
**F_43**	3	Non-truncating	2.062164019	1.28342626	3.31341236	0.00277755
**F_44**	3	Non-truncating	2.096111198	1.30140221	3.37611395	0.00233997
**F_45**	2	Non-truncating	2.114335937	1.31300972	3.40470934	0.00206764
**F_46**	1	Non-truncating	2.092782244	1.30102071	3.36638571	0.00232643
**F_47**	5	Non-truncating	2.161224211	1.34297662	3.47801296	0.00149944
**F_48**	11	Non-truncating	2.063328778	1.2812225	3.32286206	0.00288879
**F_49**	5	Non-truncating	2.043072916	1.27408925	3.27618097	0.00302288

**F_50**	14	Non-truncating	2.090640444	1.29446519	3.37651216	0.00256744
**F_51**	21	Non-truncating	2.09379454	1.29497347	3.38537869	0.00257462
**F_52**	3	Non-truncating	2.096111198	1.30140221	3.37611395	0.00233997
**F_53**	1	Non-truncating	2.13719275	1.3286532	3.43776151	0.00173768
**F_54**	1	Non-truncating	2.13719275	1.3286532	3.43776151	0.00173768
**F_55**	4	Non-truncating	2.147740279	1.33383247	3.45829661	0.00165963
**F_56**	1	Non-truncating	2.13719275	1.3286532	3.43776151	0.00173768
**F_57**	3	Non-truncating	2.062164019	1.28342626	3.31341236	0.00277755
**F_58**	1	Non-truncating	2.13719275	1.3286532	3.43776151	0.00173768
**F_59**	5	Non-truncating	2.128635187	1.32040379	3.43159252	0.00193031
**F_60**	8	Non-truncating	2.137143735	1.32501937	3.44703138	0.00184654
**F_61**	9	Non-truncating	2.099631534	1.300893	3.38878954	0.00238932
**F_62**	2	Non-truncating	2.075609106	1.29082453	3.33752038	0.00258313
**F_63**	2	Non-truncating	2.075609106	1.29082453	3.33752038	0.00258313
**F_64**	7	Non-truncating	2.099178139	1.30127772	3.38632468	0.00237064
**F_65**	3	Truncating	2.068199457	1.28537727	3.32777707	0.00274844
**F_66**	2	Truncating	2.13476731	1.32185474	3.44760382	0.00192863
**F_67**	6	Truncating	2.116580233	1.3056991	3.43104463	0.00234796
**F_68**	5	Truncating	2.089201069	1.29242469	3.37718795	0.00263939
**F_69**	2	Truncating	2.13476731	1.32185474	3.44760382	0.00192863
**F_70**	2	Truncating	2.13476731	1.32185474	3.44760382	0.00192863
**F_71**	4	Truncating	2.058387663	1.27981571	3.31060146	0.00290529
**F_72**	2	Truncating	2.080322077	1.29261343	3.3480543	0.00255139
**F_73**	3	Truncating	2.068199457	1.28537727	3.32777707	0.00274844
**F_74**	1	Truncating	2.095540847	1.30222012	3.37215757	0.00230415
**F_75**	6	Truncating	2.043665266	1.27205653	3.28331927	0.00312872
**F_76**	3	Truncating	2.068199457	1.28537727	3.32777707	0.00274844

**F_77**	9	Truncating	2.085007404	1.28679841	3.37835036	0.00284448
**F_78**	3	Truncating	2.068199457	1.28537727	3.32777707	0.00274844
**F_79**	11	Truncating	2.189479032	1.34863461	3.5545717	0.00152557
**F_80**	7	Truncating	2.225410654	1.37828204	3.59320693	0.00106586
**F_81**	14	Truncating	2.054829332	1.27124096	3.32141878	0.00328671
**F_82**	3	Truncating	2.223235429	1.38170733	3.57729577	0.00099366
**F_83**	3	Truncating	2.068199457	1.28537727	3.32777707	0.00274844
**F_84**	13	Truncating	2.018321272	1.261973	3.22797775	0.00337701
**F_85**	6	Truncating	2.157185444	1.33027057	3.49812222	0.00182663
**F_86**	7	Truncating	2.104405598	1.29813667	3.41144582	0.00253929
**F_87**	7	Truncating	2.038078046	1.2693456	3.27236502	0.00320626
**F_88**	4	Truncating	2.058387663	1.27981571	3.31060146	0.00290529
**F_89**	5	Truncating	2.089201069	1.29242469	3.37718795	0.00263939
**F_90**	4	Truncating	2.058387663	1.27981571	3.31060146	0.00290529
**F_91**	1	Truncating	2.095540847	1.30222012	3.37215757	0.00230415
**F_92**	1	Truncating	2.095540847	1.30222012	3.37215757	0.00230415
**F_93**	3	Truncating	2.11650616	1.3094911	3.42086962	0.00220798
**F_94**	1	Truncating	2.157402755	1.33820693	3.47807694	0.00160145
**F_95**	2	Truncating	2.13476731	1.32185474	3.44760382	0.00192863
**F_96**	5	Truncating	2.276887838	1.42357103	3.64169972	0.00059493
**F_97**	5	Truncating	2.130902694	1.31501745	3.45299318	0.00212647
**F_98**	2	Truncating	2.192724847	1.36037009	3.53436341	0.00126613
**F_99**	5	Truncating	2.17573216	1.34376477	3.52279695	0.00156802
**F_100**	16	Truncating	2.047290434	1.2679601	3.30562304	0.00337626
**F_101**	3	Truncating	2.223235429	1.38170733	3.57729577	0.00099366
**F_102**	1	Truncating	2.095540847	1.30222012	3.37215757	0.00230415
**F_103**	3	Truncating	2.068199457	1.28537727	3.32777707	0.00274844

**F_104**	3	Truncating	2.11650616	1.3094911	3.42086962	0.00220798
**F_105**	3	Truncating	2.168195765	1.34137851	3.50465795	0.00158435
**F_106**	3	Truncating	2.168195765	1.34137851	3.50465795	0.00158435
**F_107**	2	Truncating	2.080322077	1.29261343	3.3480543	0.00255139
**F_108**	3	Truncating	2.068199457	1.28537727	3.32777707	0.00274844
**F_109**	4	Truncating	2.19750846	1.36054694	3.54933981	0.00128782
**F_110**	4	Truncating	2.10156805	1.29993818	3.39753715	0.00244304
**F_111**	4	Truncating	2.10156805	1.29993818	3.39753715	0.00244304
**F_112**	1	Truncating	2.095540847	1.30222012	3.37215757	0.00230415
**F_113**	3	Truncating	2.11650616	1.3094911	3.42086962	0.00220798
**F_114**	1	Truncating	2.095540847	1.30222012	3.37215757	0.00230415
**F_115**	4	Truncating	2.19750846	1.36054694	3.54933981	0.00128782
**F_116**	3	Truncating	2.168195765	1.34137851	3.50465795	0.00158435
**F_117**	4	Truncating	2.250762935	1.40256475	3.61190724	0.00077396
**F_118**	1	Non-truncating	2.13719275	1.3286532	3.43776151	0.00173768
**F_119**	2	Non-truncating	2.114335937	1.31300972	3.40470934	0.00206764
**F_120**	1	Non-truncating	2.13719275	1.3286532	3.43776151	0.00173768
**F_121**	1	Non-truncating	2.13719275	1.3286532	3.43776151	0.00173768
**F_122**	2	Truncating	2.080322077	1.29261343	3.3480543	0.00255139
**F_123**	8	Truncating	2.062700454	1.27765155	3.33012013	0.00305035
**F_124**	1	Truncating	2.095540847	1.30222012	3.37215757	0.00230415
**F_125**	6	Truncating	2.043665266	1.27205653	3.28331927	0.00312872
**F_126**	2	Non-truncating	2.075609106	1.29082453	3.33752038	0.00258313
