# Supplementary material for: Nontruncating FRMD7 Variants Are More Penetrant than Truncating Variants in Heterozygous Female Carriers of Infantile Nystagmus
Source: Ophthalmol Sci. 2026 Aug 14;6(10):101363. doi: 10.1016/j.xops.2026.101363 (PMC13589131; doi:10.1016/j.xops.2026.101363)
Supplement: Supplementary file 3 [file mmc3.pdf]

Supplementary Table 3: Generalised estimating equation leave-one-PMID-out results

Removed PMID	Number of Pedigrees Removed	Number of Carriers Removed	OR	CI Lower	CI Higher	P
**21303855**	10	39	1.83565499	1.12939917	2.98355915	0.01424455
**32446246**	3	24	1.96685619	1.22497168	3.15805119	0.0051115
**24434814**	3	12	1.96831256	1.22204206	3.17031178	0.00536017
**37423457**	4	19	1.99823815	1.23102906	3.24359174	0.00509466
**22065930**	1	7	2.03807805	1.2693456	3.27236502	0.00320626
**17846367**	1	14	2.05482933	1.27124096	3.32141878	0.00328671
**24513357**	1	4	2.05838766	1.27981571	3.31060146	0.00290529
**23733424**	2	9	2.06572241	1.27988741	3.33405035	0.00297439
**30890130**	1	3	2.06819946	1.28537727	3.32777707	0.00274844
**25678693**	8	12	2.07521384	1.28009436	3.36421487	0.00305867
**18431453**	6	46	2.07991256	1.26259446	3.42630701	0.00403306
**27036142**	2	8	2.08242813	1.2962174	3.34550893	0.00242423
**20450309**	1	9	2.0850074	1.28679841	3.37835036	0.00284448
**37545716**	1	5	2.08920107	1.29242469	3.37718795	0.00263939
**34132631**	3	9	2.09065345	1.29604353	3.37244218	0.00250325
**18246032**	1	21	2.09379454	1.29497347	3.38537869	0.00257462
**33531592**	1	1	2.09554085	1.30222012	3.37215757	0.00230415
**17013395**	15	85	2.09747964	1.22749442	3.58406586	0.00673097
**23946638**	1	5	2.09808639	1.30146125	3.3823262	0.00235438
**21746984**	1	4	2.10156805	1.29993818	3.39753715	0.00244304
**31495972**	1	7	2.1044056	1.29813667	3.41144582	0.00253929
**24169426**	1	8	2.11155272	1.30839441	3.40773	0.00220775

**30618027**	1	7	2.12552336	1.31759668	3.42885621	0.0019986
**30942644**	1	5	2.12863519	1.32040379	3.43159252	0.00193031
**17962394**	1	10	2.13297881	1.32171018	3.44220593	0.00192024
**36833273**	1	2	2.13476731	1.32185474	3.44760382	0.00192863
**22490987**	1	8	2.13714374	1.32501937	3.44703138	0.00184654
**27081518**	1	1	2.13719275	1.3286532	3.43776151	0.00173768
**30015830**	1	1	2.13719275	1.3286532	3.43776151	0.00173768
**30576400**	1	5	2.16122421	1.34297662	3.47801296	0.00149944
**25916882**	1	3	2.17063235	1.3509372	3.48768604	0.00135895
**35705619**	2	19	2.17132509	1.34281654	3.51101772	0.00156572
**30025138**	4	10	2.1719759	1.34029641	3.51972838	0.00163737
**35762937**	4	10	2.21126287	1.36511579	3.58188185	0.00126075
**26268155**	2	4	2.21457432	1.36775102	3.58569603	0.00122178
**33732697**	5	9	2.22194341	1.37043105	3.60253989	0.00120333
**30616528**	1	7	2.22541065	1.37828204	3.59320693	0.00106586
**21365021**	1	8	2.22879929	1.39454124	3.56213651	0.00080774
**28378818**	3	12	2.24436683	1.37543672	3.66224225	0.00121221
**22262942**	1	5	2.27688784	1.42357103	3.64169972	0.00059493
